# Machine Learning Classification of Cirrhotic Patients with and without Minimal Hepatic Encephalopathy Based on Regional Homogeneity of Intrinsic Brain Activity

**DOI:** 10.1371/journal.pone.0151263

**Published:** 2016-03-15

**Authors:** Qiu-Feng Chen, Hua-Jun Chen, Jun Liu, Tao Sun, Qun-Tai Shen

**Affiliations:** 1 School of Information Science and Engineering, Central South University, Changsha 410083, China; 2 Department of Radiology, The First Affiliated Hospital of Nanjing Medical University, Nanjing 210029, China; Taipei Veterans General Hospital, TAIWAN

## Abstract

Machine learning-based approaches play an important role in examining functional magnetic resonance imaging (fMRI) data in a multivariate manner and extracting features predictive of group membership. This study was performed to assess the potential for measuring brain intrinsic activity to identify minimal hepatic encephalopathy (MHE) in cirrhotic patients, using the support vector machine (SVM) method. Resting-state fMRI data were acquired in 16 cirrhotic patients with MHE and 19 cirrhotic patients without MHE. The regional homogeneity (ReHo) method was used to investigate the local synchrony of intrinsic brain activity. Psychometric Hepatic Encephalopathy Score (PHES) was used to define MHE condition. SVM-classifier was then applied using leave-one-out cross-validation, to determine the discriminative ReHo-map for MHE. The discrimination map highlights a set of regions, including the prefrontal cortex, anterior cingulate cortex, anterior insular cortex, inferior parietal lobule, precentral and postcentral gyri, superior and medial temporal cortices, and middle and inferior occipital gyri. The optimized discriminative model showed total accuracy of 82.9% and sensitivity of 81.3%. Our results suggested that a combination of the SVM approach and brain intrinsic activity measurement could be helpful for detection of MHE in cirrhotic patients.

## Introduction

Minimal hepatic encephalopathy (MHE) is a neurocognitive complication of cirrhosis, which has been reported in 30%–80% of tested patients [1, 2]. As the mildest form of hepatic encephalopathy (HE), MHE is defined as a condition in which cirrhotic patients have neuropsychiatric and neurophysiological defects, despite normal mental status. MHE is characterized by a specific spectrum of neurocognitive impairments that mainly affect attention, memory, and executive abilities [3]. Notably, these neurocognitive deficits are subtle, and cannot be detected by routine clinical examinations [1], resulting in a relatively high rate of missed diagnosis and patients going untreated. As it remains challenging to detect MHE in cirrhotic patients, identification of new biomarkers or the development of novel screening methods for MHE diagnosis would be helpful for treatment and improving prognosis.

Both neurophysiological and neuroimaging studies have demonstrated that MHE is associated with abnormal neuronal activity at baseline. For example, electroencephalogram studies have revealed changes in cortical activity in cirrhotic patients with MHE [1, 4, 5]. Abnormal electrophysiological activity could be regarded as reflecting the presence of MHE [6]. Positron emission tomography studies show decreased cerebral glucose metabolism rate (another index of neuronal activity) in cirrhotic patients with MHE [7–9]. Moreover, the alteration of cerebral glucose metabolism rate is found to be correlated with impaired neurocognition in cirrhosis [10]. A study using near-infrared spectroscopy further supports the association of MHE with impaired brain activity [11].

Recent resting-state functional magnetic resonance imaging (fMRI) studies also emphasize the importance of aberrant brain intrinsic activity in the pathogenesis of MHE. For example, Chen and colleagues [12, 13] report MHE-related changes in brain regional intrinsic neuronal activity by measuring the amplitude of low-frequency fluctuations (ALFF) in fMRI signals. Similarly, regional homogeneity (ReHo), an index reflecting the local synchrony of intrinsic brain activity, is also shown to be altered in MHE patients [14]. Moreover, changes in ALFF and ReHo are correlated with the cognitive impairments seen in MHE and progress with advancement of HE [15, 16]. These findings suggest the potential of resting-state fMRI to provide biomarkers for identification of MHE. A previous study indicates that analysis of the resting-state fMRI signal, e.g., functional connectivity [17], could be helpful for detecting MHE in cirrhotic patients.

Machine learning-based approaches have been used to examine fMRI data in a multivariate manner and extract features predictive of disease-related membership. The present study was performed to use the support vector machine (SVM) method to identify MHE in cirrhotic patients based on measurement of intrinsic brain activity. The ReHo method was performed to assess the local synchrony of intrinsic brain activity. The voxel-wise ReHo value was then used as a discriminative index. The SVM classification was applied to investigate the brain discriminative ReHo maps using leave-one-out cross-validation. This study represents the first attempt to discriminate MHE in cirrhotic patients based on examination of regional homogeneity of brain intrinsic activity and machine learning. It would be helpful to verify the potential of the ReHo index as a diagnostic biomarker for MHE.

## Patients and Methods

### Subjects

This study was approved by the Research Ethics Committee of The First Affiliated Hospital of Nanjing Medical University, China, and written informed consent was obtained from all subjects. A total of 41 cirrhotic patients were enrolled in this study. Subjects with current overt HE or other neuropsychiatric disorders, and those taking psychotropic medications, suffering from uncontrolled endocrine or metabolic diseases, or with alcohol abuse within 6 months prior to the study were excluded. Six subjects were excluded: two with cerebral infarction (detected by routine MRI scanning), two with current overt HE, one with thyroid dysfunction, and one with diabetic ketoacidosis. The final study population therefore consisted of the remaining 35 subjects—16 patients with MHE and 19 patients without MHE (NHE).

### Neurocognitive tests and MHE diagnosis

The diagnosis of MHE was based on Psychometric Hepatic Encephalopathy Score (PHES) using forms kindly provided by Prof. Karin Weissenborn, Hannover Medical School, Germany. PHES includes five neurocognitive tests, i.e., digit-symbol test (DST, in numbers), number connection test A (NCT-A, in seconds), number connection test B (NCT-B, in seconds), serial dotting test (SDT, in seconds), and line tracing test (LTT, measured as the sum of the time to complete test and the error score). Due to the differences between Chinese and English alphabets, the characters in the NCT-B were replaced with Chinese characters in the same order [18, 19]. For each PHES subtest, to obtain equations for predicting results from age and education year, an additional 150 healthy subjects were included in this study. The details about calculation of PHES score were described previously [18–20]. The mean PHES in 150 healthy subjects was –0.36 ± 2.04 (range: –9 –+5). MHE was diagnosed when PHES performance was impaired by two standard deviations beyond normative performance [18–20]. Therefore, a diagnosis of MHE was made when the PHES score was ≤ –5.0 points.

### MRI data acquisition

MRI data were collected using a 3.0 T scanner (Siemens, Verio, Germany). Functional images were obtained using an echo planar imaging sequence with the following parameters: 35 contiguous axial slices, TR = 2,000 ms, TE = 25 ms, FOV = 240 mm × 240 mm, matrix = 64 × 64, flip angle = 90°, slice thickness = 4 mm. Three-dimensional T1-weighted magnetization-prepared rapid gradient echo (MPRAGE) sagittal images were collected using the following parameters: TR = 1.9 ms, TE = 2.48 ms, FOV = 256 mm × 256 mm, matrix = 256 × 256, flip angle = 9°, slice thickness = 1.0 mm, 176 slices. The participants were instructed to keep their eyes closed, “not to think of anything in particular,” and to keep their heads still during fMRI scanning.

### Resting-state fMRI data preprocessing

Functional data were preprocessed using Statistical Parametric Mapping (SPM, http://www.fil.ion.ucl.ac.uk/spm) software and the Data Processing Assistant for Resting-State fMRI (DPARSF) tool [21]. The first 10 volumes were discarded to allow for scanner calibration and adaptation of the participants to the scanning environment. The remaining volumes were then corrected for differences in slice acquisition times and were realigned to correct for head movements. Subjects with > 3 mm maximum displacement in any of the x, y, or z directions, or more than 3.0° of angular rotation about any axis, were excluded from the study. Next, all of the corrected functional data were normalized to Montreal Neurological Institute space and resampled to a 3-mm isotropic resolution. The resulting images were further temporally band-pass filtered (0.01–0.08 Hz) to reduce the effects of low-frequency drift and high-frequency physiological noise, and linear trends were also removed.

### ReHo analysis

ReHo analysis was carried out using REST software (http://restfmri.net/). Kendall’s coefficient of concordance (KCC) was used to measure the regional homogeneity of the ranked time series of a given voxel with the nearest 26 neighboring voxels. To reduce the effects of individual variability, we normalized the ReHo value of each voxel by dividing it by the mean ReHo of the whole brain for each subject. Then, the data were smoothed with a Gaussian filter of 8-mm full width at half-maximum (FWHM).

### SVM procedure

**Fig 1:**
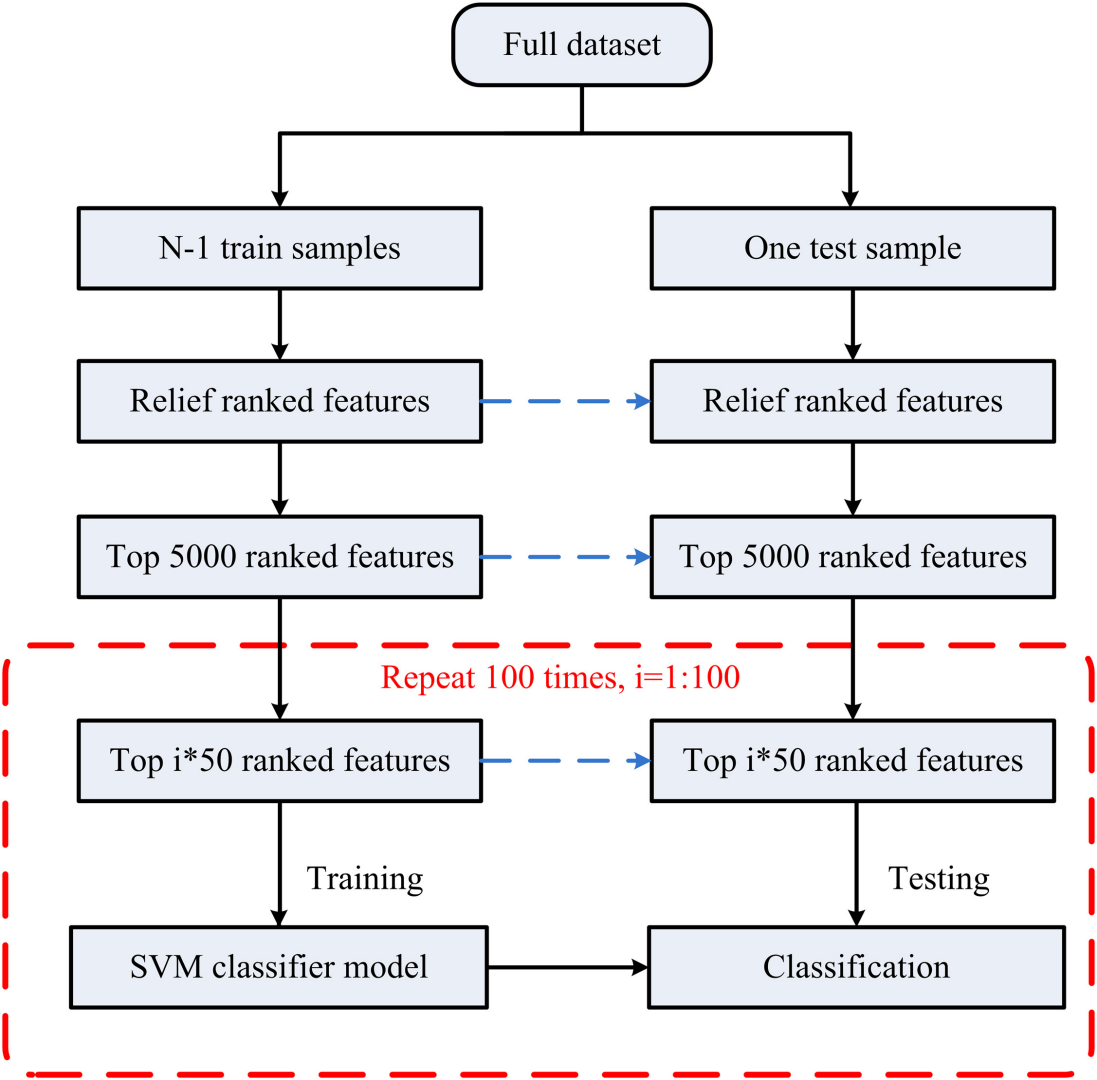
Machine learning analysis flow chart.

The SVM classification process was conducted using the LibSVM MATLAB library (http://www.csie.ntu.edu.tw/~cjlin/libsvm/). To examine SVM classifier performance, a leave-one-out cross-validation approach was applied in this study. Fig 1 shows a flow chart of machine learning analysis, including (i) dividing the samples into a training set and a test set, (ii) ranking features and selecting the most discriminative voxels, (iii) building the SVM classifier model using the training samples, and (iv) evaluating the performance of the SVM model using the test sample.

**Fig 2:**
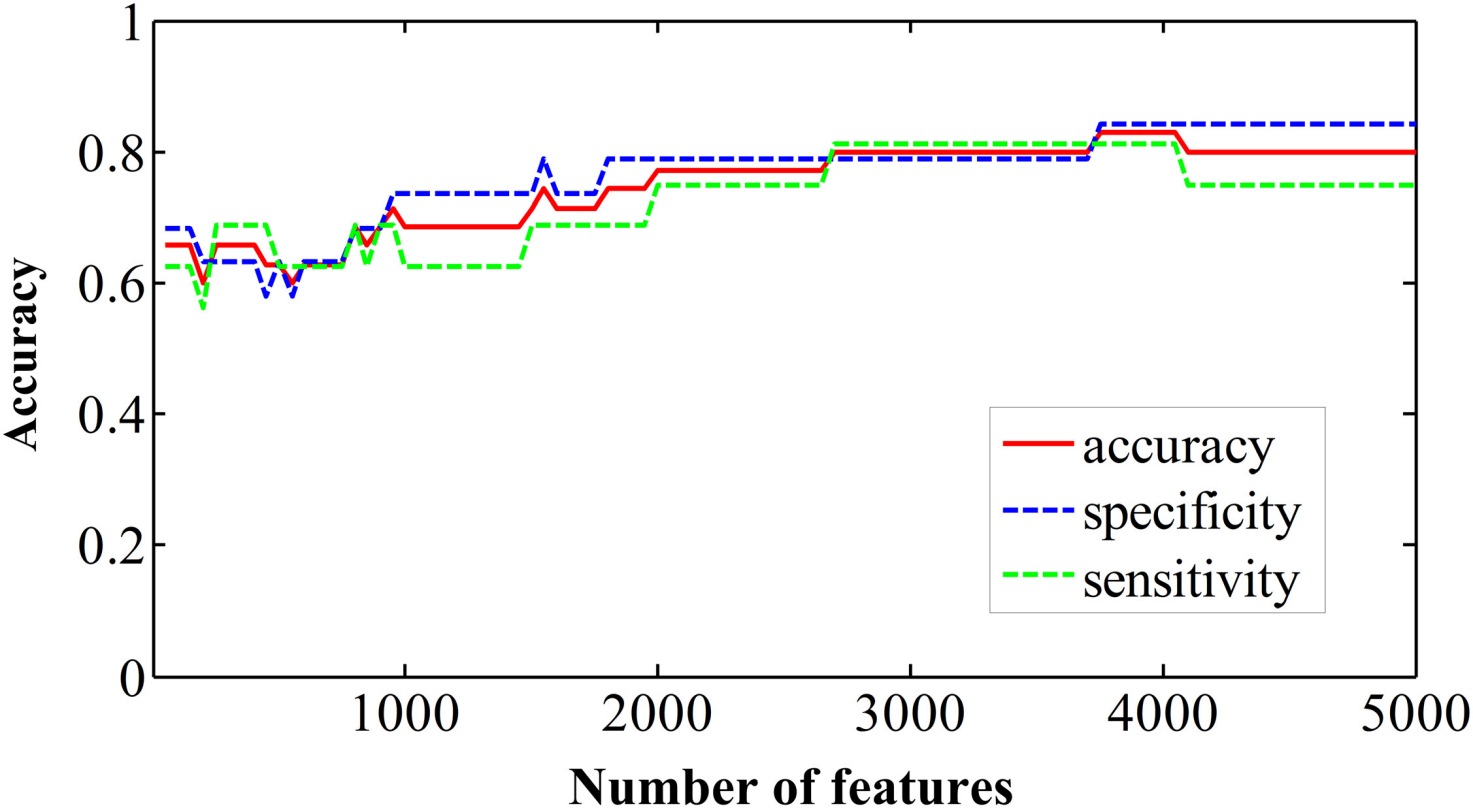
Accuracy, sensitivity, and specificity of ReHo (regional homogeneity)-based classification.

We selected features with the greatest discriminative ability and then the SVM classifier was used to solve the classification problem. The discriminative power of a feature can be measured quantitatively by its relevance to classification. In this study, feature evaluation was performed by the ReliefF feature weighting algorithm [22]. For a randomly selected sample, the “near-hit” (the nearest samples in feature space in the same class) and the “near-miss” (the nearest samples in a different class) were first determined. The weight attributed to each feature was computed from the difference in feature values between the classes and the near-hit/near-miss samples, based on the assumption that samples of the same class should be more similar to each other than to those of another class. In this study, the ReliefF algorithm was used to evaluate how well the ReHo-value of a given voxel distinguished between instances and then to automatically select the voxels with the most predictive power. This method allowed us to scale the voxel-wise discriminative power and rank the features. The top 5,000 features with most predictive power were then selected. Next, the ranked features were selected in a loop with increased length of 50. For each loop, the selected features were taken as inputs. The number of input features ranged from 50 to 5,000, resulting in an accuracy curve for ReHo-based classification. Here, we did not evaluate SVM classifier performance using a larger number of features (> 5,000), because this would have included more non-discriminative voxels into the classification model and reduced the accuracy, as shown in Fig 2. To determine the final discriminative map, a linear SVM model was applied to train and test the selected features. The weight (*w*) parameter was calculated to describe the separating hyperplane of the linear SVM and used to indicate the contribution of the feature to the SVM classifier. Within the discriminative map, positive *w* indicates higher ReHo value in MHE patients than in controls, while a negative value indicates a higher ReHo value in controls than in MHE patients [23]. In addition, to validate the binary classification of the subject groups on a quantitative level and to identify the extent to which the classification is driven by MHE symptom rather than confounding factors, we correlated the test margin (the distance from the optimal hyperplane of SVM) to the MHE diagnostic criteria-PHES result, using Pearson’s correlation analysis [23].

As the classifier may be ill-trained due to the small number of samples, but huge numbers of features, we performed permutation tests (10,000 permutations) to estimate the statistical significance of the observed classification accuracy [24, 25]. In permutation testing, the generalization rate was chosen as the statistic.

### Correlation analysis

To further validate the biomarkers detected by the SVM classifier, we performed Pearson’s correlation analysis to examine the relationships between ReHo changes and clinical characteristics, such as PHES score and Child–Pugh score, in the MHE group. The statistical map was corrected by using the AlphaSim program, with the threshold set at *P* < 0.05 determined by Monte Carlo simulations [26] (Parameters were single voxel *P* = 0.01, a minimum cluster size of 15 voxels, 5000 simulations, FWHM = 8 mm, with mask). We restricted this voxel-wise correlation analyses to the final discriminative ReHo-map that was identified by the SVM method. Age and education level were included as covariates.

## Results

The demographic and clinical characteristics of the subjects are summarized in Table 1. No significant differences were found with regard to age, gender, or education level between the two groups. MHE patients had significantly poorer neurocognitive performance compared to NHE patients.

**Table 1:** Demographic and clinical characteristics of cirrhotic patients.

Characteristic	NHE patients (*n* = 19)	MHE patients (*n* = 16)	*P*-value
Age (years)	51.2 ± 10.0	50.1 ± 9.9	0.74
Sex (male/female)	15/4	13/3	0.87 (χ^2^ test)
Education (year)	8.2 ± 3.1	8.1 ± 2.1	0.93
Etiology of cirrhosis (HBV/alcoholism/HBV + alcoholism/other)	12/2/2/3	9/3/1/3	–
Previous episode of overt HE (yes/no)	5/14	9/7	0.072
Child–Pugh stage (A/B/C)	14/5/0	4/9/3	–
Digit symbol test (raw score)	42.1 ± 11.7	29.3 ± 9.1	0.001
Number connection test A (seconds)	39.1 ± 11.0	56.2 ± 12.3	< 0.001
Number connection test B (seconds)	73.3 ± 19.9	110.9 ± 40.9	0.001
Serial dotting test (seconds)	45.2 ± 9.2	57.3 ± 8.7	< 0.001
Line tracing test (raw score)	150.4 ± 39.0	188.9 ± 39.6	0.017
PHES score	–0.6 ± 2.0	–7.0 ± 2.0	< 0.001

Abbreviation: NHE, no hepatic encephalopathy; MHE, minimal hepatic encephalopathy; HBV, hepatitis B virus; HE, hepatic encephalopathy; PHES, Psychometric Hepatic Encephalopathy Score.

The typical ReHo patterns were found in two groups (see S1 Fig). Fig 2 shows the sensitivity, specificity, and accuracy of ReHo-based classification. The highest accuracy was continually obtained when 3,750–4,050 features were selected. The optimized accuracy, sensitivity, and specificity were 82.9%, 81.3%, and 84.2%, respectively.

**Fig 3:**
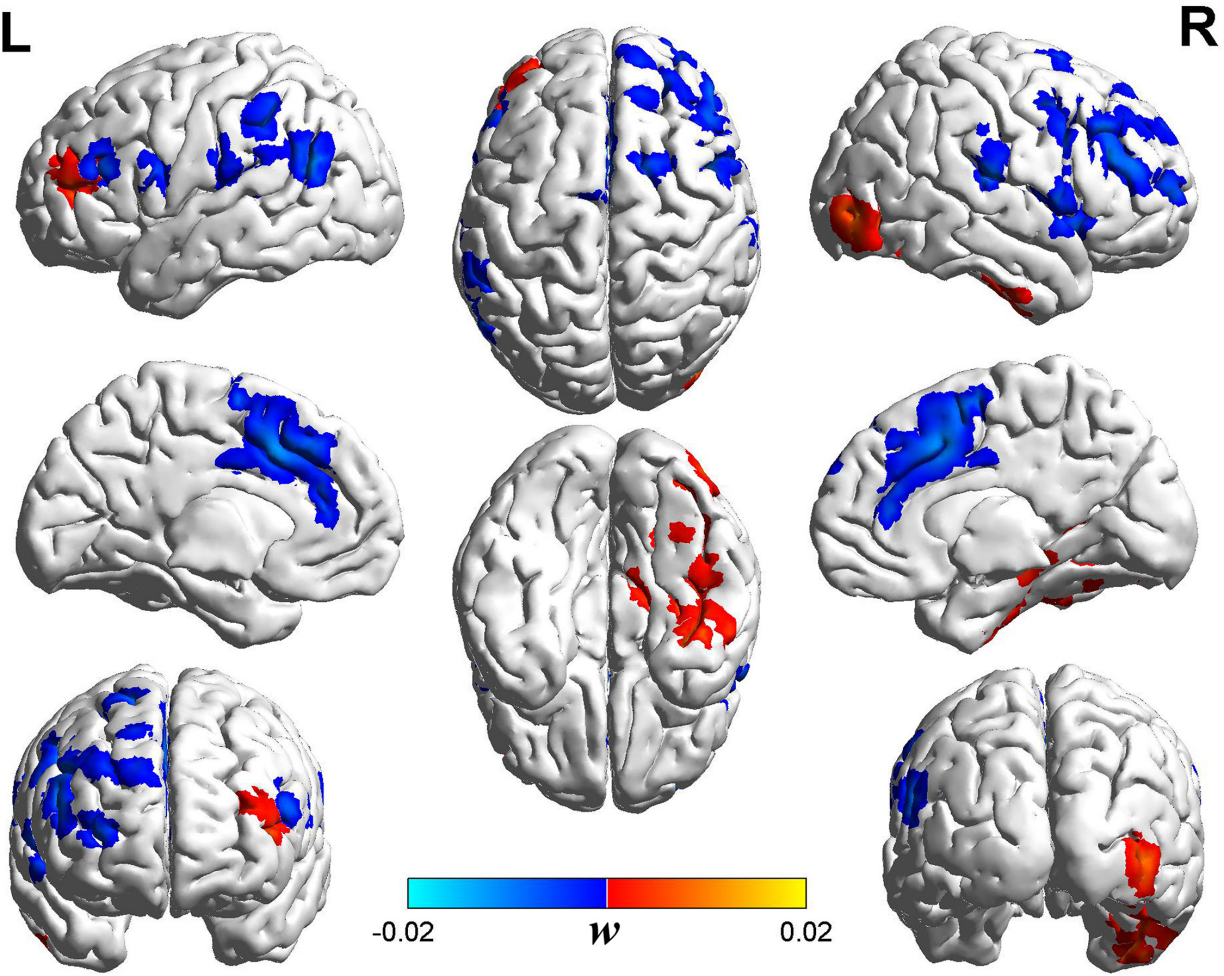
Discriminative ReHo-map for minimal hepatic encephalopathy (MHE) subjects. The map is composed of 4,000 features as the optimized classification performance is achieved. The color intensity indicates the attribute weight of a feature in support vector machine (SVM) classification. The map shown includes clusters with > 50 voxels. The positive and negative weights indicate relatively increased and decreased ReHo values, respectively, in MHE patients as compared to NHE group.

**Fig 4:**
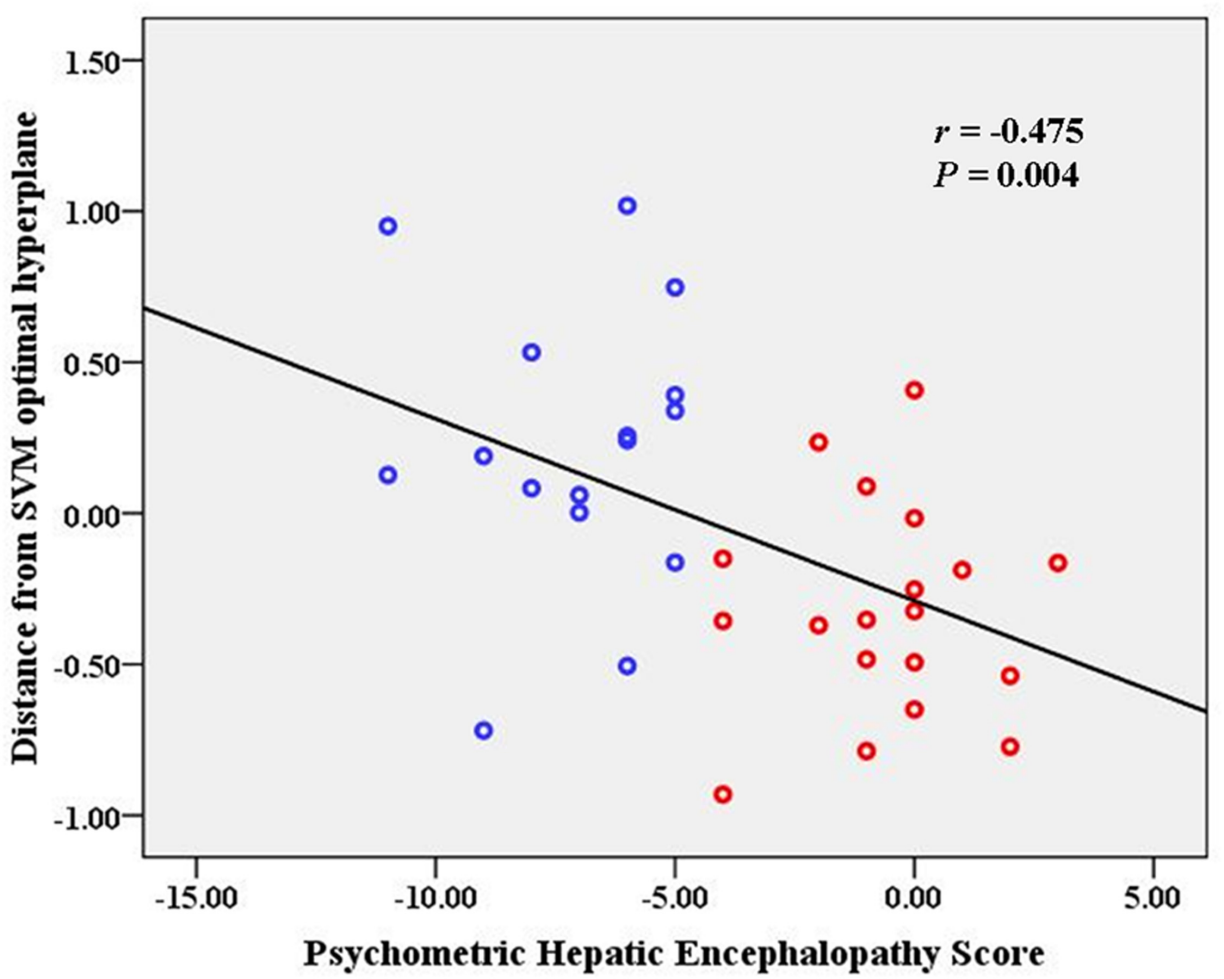
Correlation between test margin (i.e. the distance from SVM optimal hyperplane) and diagnostic criteria. The blue and red circles indicate the subjects without and with MHE, respectively.

Fig 3 shows the discriminative ReHo map consisting of 4,000 features. The color intensity indicates the attribute weight (*w*) for SVM classification. The positive and negative weights indicate relatively increased and decreased ReHo values in MHE patients as compared to the NHE group, respectively [23]. The most important regions discriminating between MHE and NHE subjects are summarized in Table 2, and included the prefrontal cortex, anterior cingulate cortex, anterior insular cortex, inferior parietal lobule, precentral and postcentral gyri, superior and medial temporal cortices, and middle and inferior occipital gyri. Notably, a discriminative ReHo map could be obtained in each leave-one-out cross-validation. However, the space distributions of these ReHo-maps were very similar, so we chose one of the ReHo maps at random to represent the final discriminative map (Fig 3). Across the whole subject group (including both MHE and NHE patients), the test margin was negatively correlated with the PHES result(r = -0.475, *P* = 0.004) (Fig 4).

**Table 2:** Most important regions discriminating between MHE and NHE subjects.

Clusters	Hemisphere	Brain regions	Custer size (voxel)	BA	MNI coordinates (x, y, z)			Peak *w* value
1	Right	Inferior frontal gyrus	137	47/13/6/22/44/38	60	3	6	–0.012
		Insular cortex						
		Superior temporal gyrus						
		Precentral gyrus						
2	Left	Supramarginal gyrus	168	40/13/41	–39	–27	15	–0.019
		Insular cortex						
		Superior temporal gyrus						
		Postcentral gyrus						
3	Right	Supramarginal gyrus	94	40/43/2/4	63	–18	15	–0.014
		Postcentral gyrus						
		Precentral gyrus						
		Superior temporal gyrus						
4	Left	Inferior frontal gyrus	53	44/6	–57	9	21	–0.007
		Precentral gyrus						
5	Bilateral	Medial frontal gyrus	638	32/6/8/24/9	3	15	42	–0.013
		Anterior cingulate cortex						
		Superior frontal gyrus						
6	Right	Middle frontal gyrus	558	46/10/9	51	9	36	–0.016
		Superior frontal gyrus						
		Inferior frontal gyrus						
7	Left	Middle frontal gyrus	86	46/10	–51	33	21	–0.008
		Inferior frontal gyrus						
8	Left	Supermarginal gyrus	91	39/40/22	–54	–63	18	–0.013
		Angular gyrus						
		Superior temporal gyrus						
9	Right	Supermarginal gyrus	148	40	–33	–42	39	–0.016
10	Right	Inferior occipital gyrus	587	20/19/37/18	45	–3	–42	0.018
		Inferior temporal gyrus						
		Parahippocampal gyrus						
		Fusiform gyrus						
		Middle occipital gyrus						
11	Left	Middle frontal gyrus	115	10/46	–42	45	6	0.008
		Inferior frontal gyrus						

Abbreviation: MHE, minimal hepatic encephalopathy; NHE, no hepatic encephalopathy; BA, Brodmann area; MNI, Montreal Neurological Institute; *w*, attribute weight for the peak voxel.

**Fig 5:**
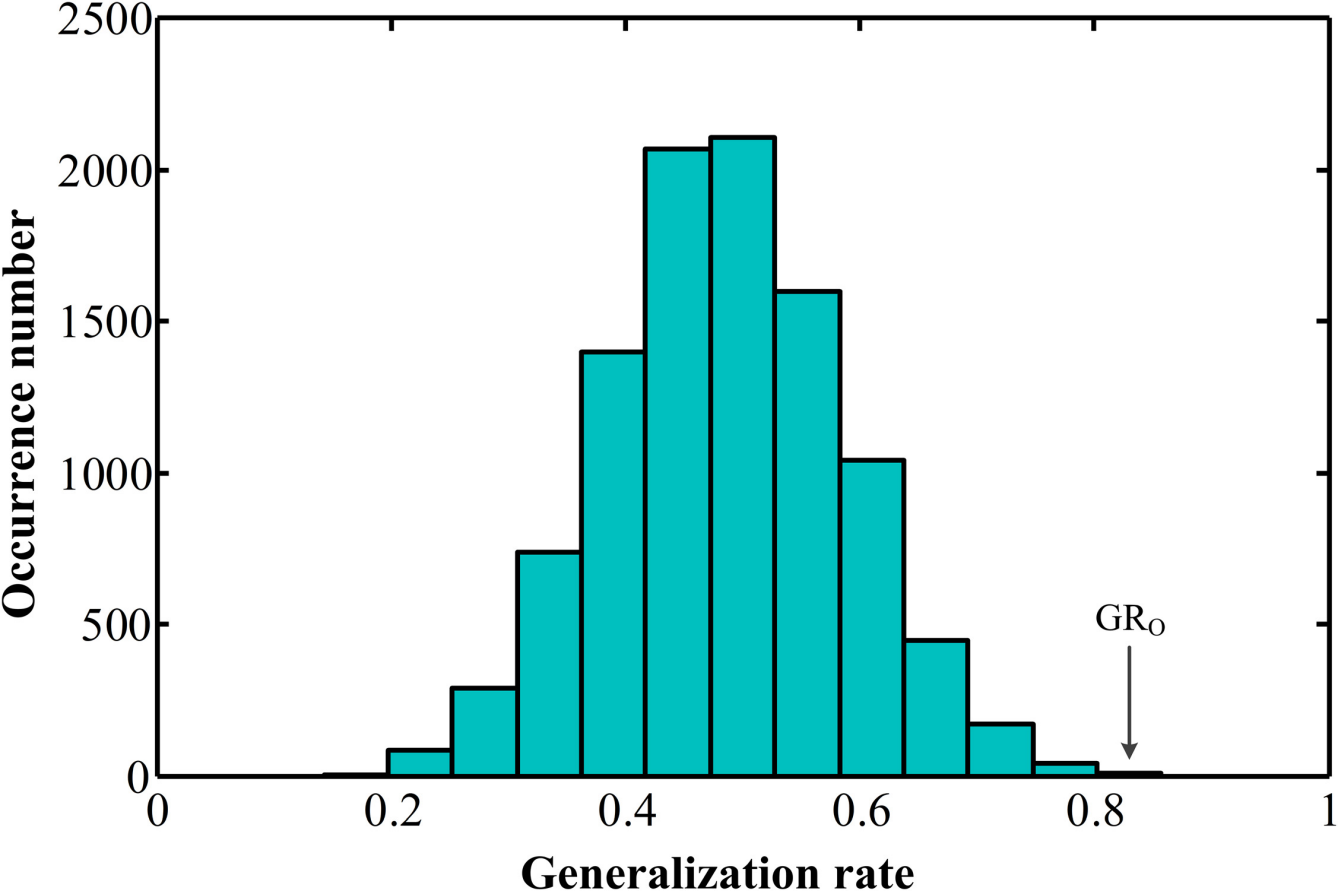
Permutation distribution of the estimate (repetitions: 10,000) as the 4,000 most discriminating features were used in the linear support vector machine classifier. GR_0_ is the generalization rate obtained by the classifier trained on the real class labels. With the generalization rate as the statistic, the classifier learned the relationship between the data and labels with a probability of being wrong < 0.0005.

Furthermore, the statistical significance of the observed classification accuracies was estimated by permutation testing, using the generalization rate as the statistic. Fig 5 shows the permutation distribution of the estimate, as the 4,000 most discriminating features were used in the classifier. The classifier learned the relationship between the data and the labels with a probability of being wrong < 0.0005, indicating the reliability of our classification results.

**Fig 6:**
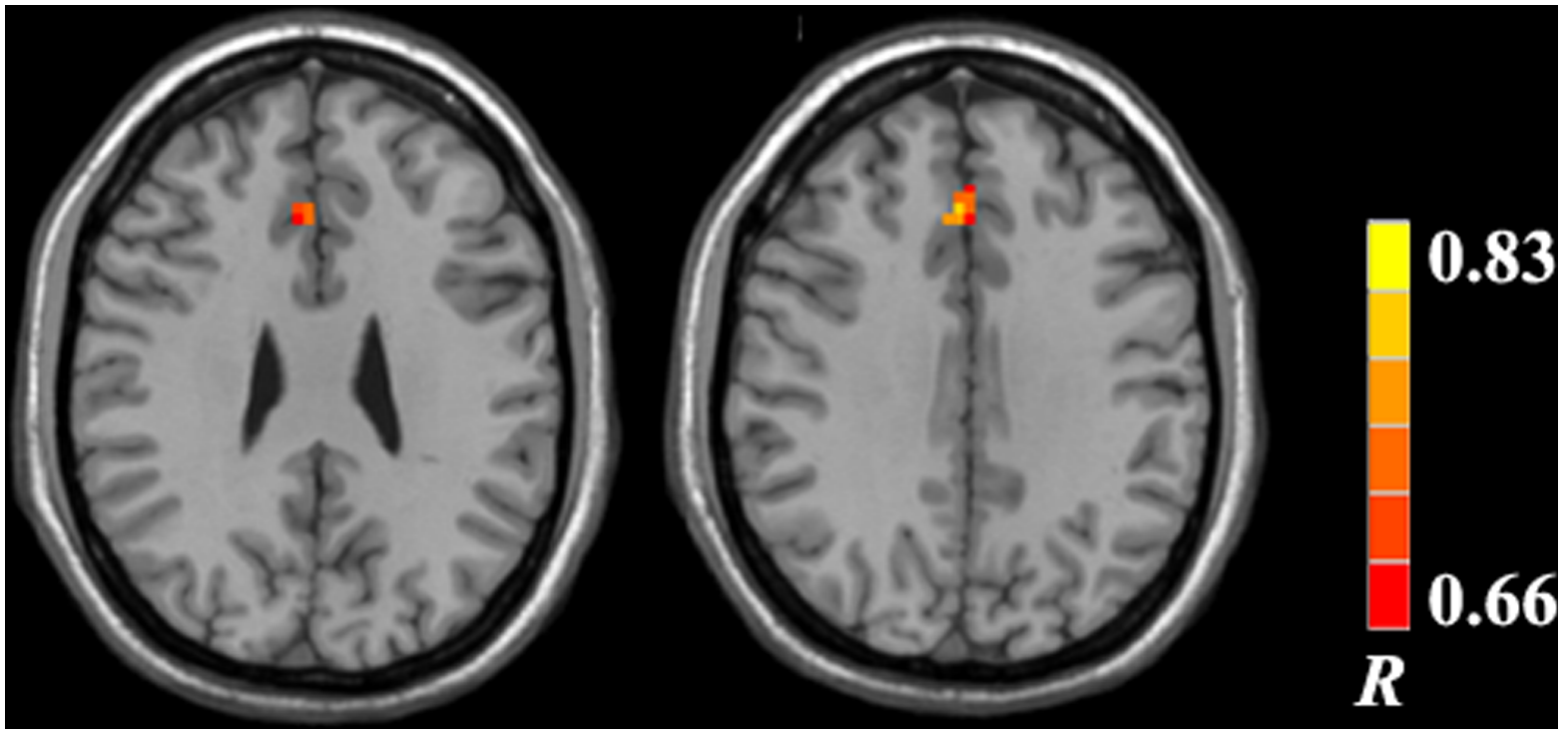
Correlation map of ReHo value and Psychometric Hepatic Encephalopathy Score (PHES) in the MHE group. Significant positive correlations were found in the anterior cingulate gyrus and medial prefrontal cortex.

As shown in Fig 6 and S2 Fig, the ReHo values in the anterior cingulate gyrus and medial prefrontal cortex were positively correlated with PHES score in the MHE group. No significant correlation was found between ReHo change and Child–Pugh score.

## Discussion

In this study, we applied a machine learning method to search the discriminative ReHo-map for MHE among cirrhotic patients. To the best of our knowledge, this was the first attempt to create an optimized discriminative ReHo-map for MHE, based on an automated procedure that combines resting-state fMRI with an SVM-classification method. Previous studies have documented that MHE is a neuropathological condition characterized by abnormal intrinsic brain activity [15, 27, 28]. The discriminative map highlights a set of regions in which regional synchrony of brain activity could be used as classification features, such as the prefrontal cortex, anterior cingulate cortex, anterior insular cortex, inferior parietal lobule, precentral and postcentral gyri, superior and medial temporal cortices, and middle and inferior occipital gyri. Our result was consistent with existing findings, because the pattern of neural activity in these brain regions has been demonstrated to be affected by early HE [15, 16, 18, 27, 28]. This coherence validated the clinical significance of ReHo-feature selection in the current study. Our method yielded a high degree of classification accuracy, which indicated the usefulness of SVM-approach for identifying biomarker of MHE. The relationship between ReHo-value from the discriminative map with PHES score (the clinical marker of MHE) further confirmed the utility of these potential biomarkers for diagnosing MHE.

Our results could have practical significance, because early detection of MHE is helpful for physicians given that MHE is associated with poor prognoses [29, 30] but can be reversed by prompt treatment [31]. A new neuroimaging biomarker would contribute to early diagnosis and therapy for MHE. In addition, as the potential biomarker, the ReHo-metric may provide supplementary information for routine MHE-related neurocognitive tests. For example, psychometric tests (e.g. PHES [1]) are currently considered as the standards for diagnosing MHE; however, the version of these tests used and the interpretation of their results are not very consistent across the countries with different cultures and official languages. The application of a neuroimaging biomarker could improve the international comparability of MHE-related research data.

The bilateral anterior cingulate cortex and medial frontal cortex are proposed to be the pathophysiological nodes of MHE. For example, metabolic changes in the anterior cingulate cortex have been found in cirrhosis on MRS examination [32]. Also, disrupted resting-state functional connectivity in the anterior cingulate cortex has been reported in MHE and is associated with HE progression [33]. Moreover, a previous study demonstrates decreased ReHo in the bilateral anterior cingulate cortex due to MHE [16]. These abnormalities, consistently reported in the anterior cingulate cortex, may account for the attention deficits seen in MHE, as the anterior cingulate cortex is a critical node in human attention processing [34]. In fact, attention deficit is one of the early manifestations of MHE [3]. Therefore, it is unsurprising that the anterior cingulate cortex was determined as a discriminative index by our automated feature selection and SVM classifier procedure. On the other hand, previous resting-state fMRI studies consistently indicate that MHE patients develop abnormal intrinsic brain activity in the medial frontal cortex [15, 16]. Consistent with these findings, this region was also involved in the discriminative ReHo map. The medial frontal cortex plays a necessary role in cognitive control, e.g., error prediction [35], in humans. Aberrant activity in the medial frontal cortex may reflect a neural mechanism of conflict monitoring impairment in the early stage of HE.

Impairment of cognitive control is a key characteristic of MHE patients, and is considered to be associated with abnormal activity in the anterior cingulate cortex–prefrontal cortex–parietal lobe–temporal fusion gyrus network [36]. Notably, these areas were also involved in the discriminative ReHo map. Consistent with our results, a previous study indicate that it is helpful to screen for MHE among cirrhotic patients by examining cognitive control function [37].

The ReHo feature in the anterior insular cortex was included in our SVM classification model. A previous study demonstrates abnormal intrinsic activity in the anterior insula and adjacent inferior frontal gyrus in MHE patients [15]. The anterior insular cortex is critically involved in task initiation, maintenance of attention, and performance monitoring [38, 39]. For example, as the key component of the salience network, the anterior insula cortex has been suggested to switch between the default mode network (DMN) and central executive network (CEN). DMN is characterized by deactivation during attention-demanding tasks, which plays an essential role in reallocating neuronal resources toward behavior-related processes, while CEN is associated with focused attention on the external environment during demanding cognitive tasks [40]. Therefore, abnormal intrinsic activity in the anterior insula cortex may be another mechanism underlying attention deficit and impaired executive function in MHE. This may explain why ReHo in these areas was detected by our algorithm as a classification feature.

In addition, previous studies indicate that MHE patients could have decreased ReHo and ALFF in the precentral and postcentral gyri [16, 18, 28], suggesting abnormal intrinsic activity patterns in these areas. Consistent with these observations, ReHos in the above regions were selected as classification features in the present study, further confirming the reliability of our results.

Deficits in visual and memory processing are also regarded as characteristics of MHE [3, 41]. Relatively higher ReHo values were found in the occipital gyrus and medial temporal cortex, which are regions engaged in vision and memory processing, respectively. Consistent with our findings, previous fMRI studies indicate that MHE patients could have increased functional connectivity in the intrinsic visual network [42] and increased ReHo in the parahippocampal gyrus [43]. In these studies, the increased functional connectivity and functional activity are considered as a procedure for compensatory mechanisms. Following the previous studies, we speculated that increased ReHo in the occipital gyrus and medial temporal cortex represented compensation processing, which could be beneficial for reducing the symptoms in MHE, such as visual dysfunction and impaired memory.

The present study had some limitations. First, our results were limited to a small number of cirrhotic patients. Further studies with larger datasets are recommended to validate the generality of our results. Second, mild heterogeneity, in terms of patients’ etiology and history of overt HE, may have produced bias in the classification results as different etiologies of cirrhosis and previous overt HE can induce various degrees of cerebral functional and structural impairments [44, 45]. Third, we only examined the potential of altered regional brain intrinsic activity in discriminating between NHE and MHE groups. However, an abnormal time course coefficient across distinct brain regions (e.g., functional connectivity) has been regarded as another characteristic of MHE [42, 45] and may also be useful in identifying MHE. Further studies are required to test these possibilities.

In summary, using a machine learning method, we identified a discriminative ReHo map for MHE in cirrhotic patients and obtained good classification accuracy. Our findings suggest that an altered regional intrinsic brain activity pattern may be a useful biomarker for MHE diagnosis in cirrhotic patients.

## Supporting Information

S1 Fig Within-group ReHo maps from patients without (A) and with (B) MHE. The ReHo maps were obtained by one-sample t-test. The statistical threshold was set at *P* < 0.05 (corrected by False Discovery Rate (FDR) procedure).
(TIF)

S2 Fig The average ReHo value in the anterior cingulate gyrus and medial prefrontal cortex in two groups (A), and the correlation between ReHo value from the anterior cingulate gyrus and medial prefrontal cortex and Psychometric Hepatic Encephalopathy Score in MHE group (B). (TIF)
