# Supplementary figures and images for: Machine Learning Classification of Cirrhotic Patients with and without Minimal Hepatic Encephalopathy Based on Regional Homogeneity of Intrinsic Brain Activity
Source: PLoS One. 2016 Mar 15;11(3):e0151263. doi: 10.1371/journal.pone.0151263 (PMC4792397; doi:10.1371/journal.pone.0151263)

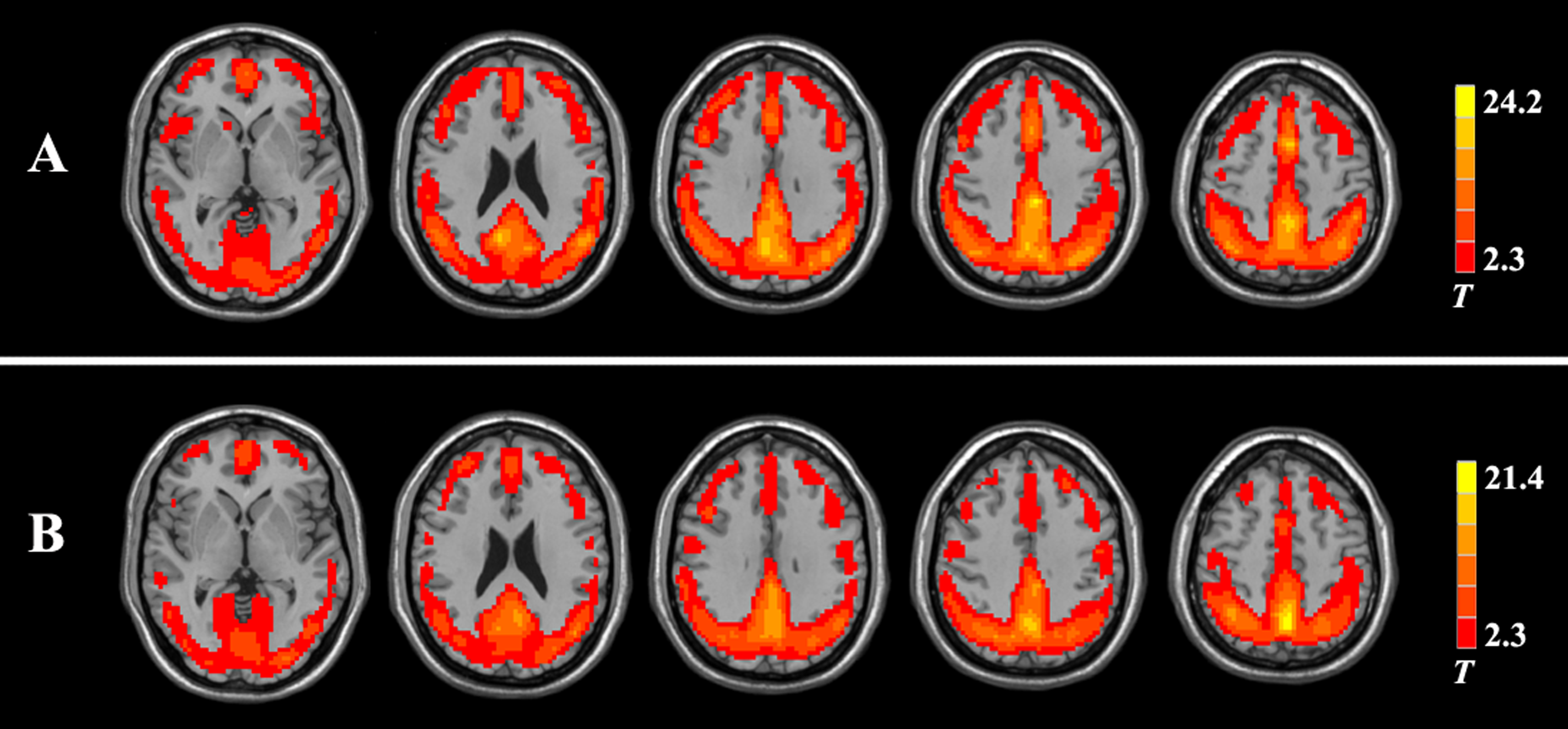

Supplement: S1 Fig — The ReHo maps were obtained by one-sample t-test. The statistical threshold was set at P < 0.05 (corrected by False Discovery Rate (FDR) procedure). (TIF) [file pone.0151263.s001.tif]

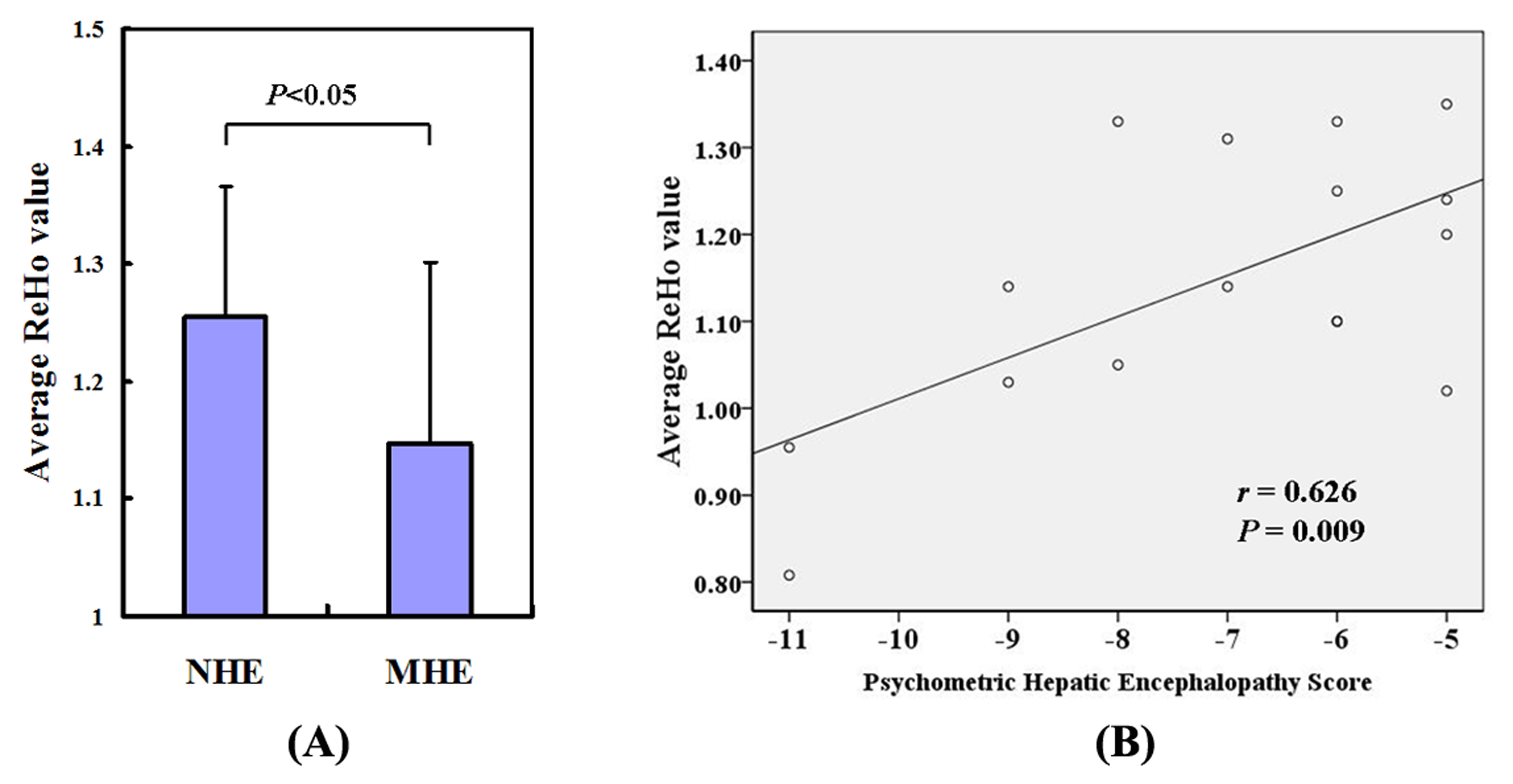

Supplement: S2 Fig — (TIF) [file pone.0151263.s002.tif]
